# Introduction of rotavirus vaccination in Palestine: An evaluation of the costs, impact, and cost-effectiveness of ROTARIX and ROTAVAC

**DOI:** 10.1371/journal.pone.0228506

**Published:** 2020-02-05

**Authors:** Frédéric Debellut, Samer Jaber, Yaser Bouzya, Jehad Sabbah, Mustafa Barham, Fakhr Abu-Awwad, Diaa Hjaija, Assad Ramlawi, Clint Pecenka, Andrew Clark, Mercy Mvundura

**Affiliations:** 1 PATH, Geneva, Switzerland; 2 Palestinian Ministry of Health, Nablus, West Bank; 3 Palestinian Ministry of Health, Ramallah, West Bank; 4 United Nations Relief and Works Agency for Palestine Refugees in the Middle East, East Jerusalem, Palestinian Territory; 5 Rostropovich-Vishnevskaya Foundation, Ramallah, West Bank; 6 Rostropovich-Vishnevskaya Foundation, Gaza City, Gaza; 7 PATH, Seattle, Washington, United States of America; 8 London School of Hygiene and Tropical Medicine, London, United Kingdom; Ministry of Health and Sports, MYANMAR

## Abstract

**Introduction:**

The Palestinian Ministry of Health (MOH) started a routine rotavirus immunization program with ROTARIX in May 2016, with support for vaccine procurement and introduction provided through a global development organization. In 2018, financial responsibility for rotavirus vaccine procurement was transferred to the Palestinian government, which elected to shift to ROTAVAC vaccine because of its lower price per dose. This study aims to assess the cost, impact, and cost-effectiveness of rotavirus vaccination, specifically evaluating the economic implications of the change in vaccine product, accounting for the different characteristics of each rotavirus vaccine used.

**Methods:**

We conducted primary and secondary data collection to assess the introduction, procurement, supply chain, and service delivery costs related to each vaccine. We used the UNIVAC model to project costs and benefits of rotavirus vaccination over a 10-year period comparing the use of ROTARIX versus no vaccination; ROTAVAC versus no vaccination; and ROTAVAC versus ROTARIX. We undertook scenario and probabilistic analyses to capture uncertainty in some of the study parameters. We used a 3% discount rate, and all costs are in 2018 US$.

**Results:**

The cost to deliver one dose was lower for ROTAVAC than ROTARIX (US$2.36 versus $2.70), but the total cost per course, excluding vaccine cost, favored ROTARIX ($7.09 versus $5.39). Both vaccines had high probability of being cost-effective interventions in Palestine compared to no vaccine. Because of lower vaccination program costs for ROTAVAC, however, switching from ROTARIX to ROTAVAC was cost-saving.

**Conclusion:**

National decision-makers should consider systematically assessing multiple criteria beyond vaccine price when comparing the health and economic value of several products in order to fully account for all characteristics including product presentation, number of doses per course, cold chain volume, cost of delivery, and wastage.

## Introduction

Despite a decreasing trend in diarrhea mortality over the 2005 to 2015 period, rotavirus remains responsible for 200,000 deaths in children under five years of age annually and millions of cases of illness requiring treatment and care [1–3]. The World Health Organization (WHO) recommends rotavirus vaccination in all national immunization programs and the vaccine is currently used by more than 95 countries worldwide [4,5]. For almost a decade, countries had to choose between two available rotavirus vaccines, ROTARIX^®^ (a registered trademark of GlaxoSmithKline Biologicals SA, used under license by GlaxoSmithKline Inc.) and RotaTeq^®^ (a registered trademark of Merck & Co., Inc.). In 2018, WHO prequalified two additional rotavirus vaccines, ROTAVAC^®^ (Bharat Biotech International Limited) and ROTASIIL^®^ (Serum Institute of India), enabling procurement by international agencies and broadening country choice [6].

The Middle Eastern and North African region has experienced a persistent burden of rotavirus infection, responsible for up to 45% of diarrhea-related hospitalizations in the region between 2010 and 2015 [7,8]. Before rotavirus vaccine introduction, the Palestinian territories of Gaza and West Bank reported 25% prevalence rates of diarrheal illnesses among children younger than six months of age, the highest in the region [9]. As of 2018, 14 out of the 22 countries of the WHO Eastern Mediterranean Region (EMRO) had introduced rotavirus vaccine [5]. The Palestinian Ministry of Health (MOH) started its routine rotavirus immunization program in May 2016, with support for vaccine procurement and introduction provided through the Rostropovich-Vishnevskaya Foundation (RVF), a global development organization. After one year of program implementation, 97.4% of the targeted birth cohort had been immunized with two doses of ROTARIX. Over the period 2015 to 2017, outpatient cases of diarrhea in children under five in Gaza dropped by 27%, while the prevalence of rotavirus in diarrheal stool samples tested at a large pediatric hospital in Bethlehem, West Bank, decreased by 65% [10].

In 2018, financial responsibility for rotavirus vaccine procurement was transferred to the Palestinian government, which elected to shift to ROTAVAC vaccine because of its lower price per dose. While ROTAVAC’s lower price per dose was a key driver in this decision, this consideration has to be balanced against other characteristics, such as the need for three doses for a complete immunization regimen with ROTAVAC rather than two with ROTARIX. ROTAVAC also comes in 5- and 10-dose vials compared to the single-dose vial for ROTARIX, leading to a likelihood of higher wastage rates with ROTAVAC. In addition, this balance must account for any potential cost implications of delivering the vaccine due to differences in presentation, storage conditions, and stability compared to ROTARIX. As shown previously, because of differing product characteristics, the choice of rotavirus vaccine product has several implications, particularly in terms of budget impact, cost, and cost-effectiveness [11].

This study aims to assess the cost, impact, and cost-effectiveness of rotavirus vaccination in Palestine, specifically evaluating the economic implications of the change in vaccine product. The primary differences examined between products includes the number of doses, price per dose, wastage, in-country logistics and the health system cost per dose. This information can be used by other countries that may be considering a product switch and other stakeholders interested in the cost implications of such a switch.

## Materials and methods

We projected costs and benefits of rotavirus vaccination over a 10-year period comparing the use of ROTARIX versus no vaccination and the use of ROTAVAC versus no vaccination. Further, we conducted a head-to-head comparison of the costs and benefits of the switch (ROTAVAC versus ROTARIX). In this analysis, we focused on the 5-dose rather than the 10-dose presentation of ROTAVAC, as this was Palestine’s preferred choice. We calculated our main study outcome, the incremental cost-effectiveness ratio, which represents the cost required to avert one disability-adjusted life-year (DALY). We used a 3% discount rate, and all costs are in 2018 US$.

To measure the benefits of rotavirus vaccination, we calculated outcomes such as the number of rotavirus cases, outpatient visits, hospitalizations, deaths, and DALYs averted by vaccination and also the same outcomes without vaccination. Our population of interest was children under five years old. When infected with rotavirus, sick children can experience a non-severe or a severe episode of rotavirus gastroenteritis (RVGE). We assumed children with non-severe episodes will either seek treatment in an outpatient setting or not, and will then recover. In contrast, children with severe episodes could seek treatment in an outpatient and/or inpatient setting or not, and would then either recover or die. Incidence rates for each disease event were applied to the under-five population to reflect the burden of rotavirus disease.

### Model

We used the UNIVAC model version 1.3.41 (www.paho.org/provac-toolkit). UNIVAC is an excel-based static cohort model developed through a collaboration between the Pan American Health Organization and the London School of Hygiene and Tropical Medicine. UNIVAC is a follow-on to the TRIVAC tool, the methods of which have been described elsewhere [12]. UNIVAC generates transparent and conservative estimates of projected impact and cost-effectiveness of new vaccines based on parameters such as population, disease incidence, vaccine schedule, efficacy, and coverage, healthcare costs and vaccine program costs. More details on UNIVAC are available elsewhere [13]. UNIVAC was developed to assist decision makers by generating evidence around the use of new vaccines, including rotavirus vaccines, and has been used in several cost-effectiveness studies to date [14,15].

### Burden of disease

We assumed an overall incidence of rotavirus gastroenteritis in the under-five Palestinian population of 10,000 cases per 100,000 per year based on a systematic review and meta-analysis [16]. We differentiated non-severe and severe episodes using results from another systematic review and meta-analysis, applying proportions of severe and moderate diarrhea as reported for EMRO [17]. To estimate the number of cases that receive treatment through an outpatient visit or hospitalization, we made additional assumptions around treatment-seeking rates. We assumed that 52.9% of cases would seek treatment in an outpatient setting based on the latest Palestine Multiple Indicator Cluster Survey (MICS) [18] and that a larger proportion of severe cases would seek treatment in an inpatient setting (80%) based on expert opinion. Finally, we used a recently published global review for disease event age distribution and a rotavirus-related mortality rate of 2.03 deaths per 100,000 per year in children under five years, which is the median value obtained from three different sources estimating rotavirus deaths [2,19,20]. DALY calculations are based on standard DALY weights and a duration of illness of three and seven days for non-severe and severe rotavirus episodes, respectively [21,22]. All parameters and input values used to model disease burden are displayed in Table 1 along with the low and high inputs used in sensitivity analyses.

**Table 1:** Study input parameters.

**Rotavirus disease burden inputs**				
	**Base case value**	**Low input**	**High input**	**Source**
Annual incidence rates per 100,000 in under-5 population				
Overall RVGE incidence	10,000	7,000	14,000	[16]
RVGE non-severe cases	8,224	6,160	11,373	[17]
RVGE non-severe visits	4,350	3,259	6,016	[18]
RVGE severe cases	1,776	839	2,627	[17]
RVGE severe visits	939.5	444	1,390	[18]
RVGE severe hospitalizations	1,421	555	2,102	Assumption
Severe RVGE deaths	2.03	0.84	4.88	[2,19,20]
Disease event age distribution				
<1 month	0%	-	-	[19]
<2 months	1%			
<3 months	3%			
<6 months	14%			
<1 year	48%			
<2 years	89%			
<3 years	98%			
<4 years	100%			
<5 years	100%			
DALY calculation				
Non-severe RVGE				
DALY weight	18.8%	12.5%	26.4	[21]
Duration of illness	3 days	-	-	[22]
Severe RVGE				
DALY weight	24.7%	16.4%	34.8%	[21]
Duration of illness	7 days	-	-	[22]
**Vaccine efficacy, coverage, and timeliness**				
	**Base case value**	**Low input**	**High input**	**Source**
Dose 1 vaccine efficacy				
2 weeks after vaccination	57.8%	45%	74%	[23]
6 months after vaccination	48.6%			
12 months after vaccination	44%			
Dose 2 vaccine efficacy				
2 weeks after vaccination	91.4%	89.9%	92.7%	[23]
6 months after vaccination	76.8%			
12 months after vaccination	69.5%			
Dose 3 vaccine efficacy				
2 weeks after vaccination	91.4%	89.9%	92.7%	[23]
6 months after vaccination	76.8%			
12 months after vaccination	69.5%			
Vaccine coverage				
Dose 1	99.5%	-	-	[24]
Dose 2	99.5%	-	-	
Dose 3	97.4%	-	-	
Coverage timeliness				
Dose 1				[19]
Coverage at 1 month	0%	-	-	
Coverage at 3 months	21%	-	-	
Coverage at 6 months	97%	-	-	
Coverage at 12 months	99.5%	-	-	
Dose 2				
Coverage at 1 month	0%	-	-	
Coverage at 3 months	0%	-	-	
Coverage at 6 months	99.5%	-	-	
Coverage at 12 months	99.5%	-	-	
Dose 3				
Coverage at 1 month	0%	-	-	
Coverage at 3 months	0%	-	-	
Coverage at 6 months	15%	-	-	
Coverage at 12 months	97.4%	-	-	
**Vaccine and commodities costs**				
	**Base case value**	**Low input**	**High input**	**Source**
ROTAVAC vaccine price	$1	$0.85	$1.5	[31, 32]
ROTARIX vaccine price	$4	-	-	[32]
Safety box unit price (100 dose volume)	$0.64	-	-	[32]
International handling (as a % of vaccine price)	3.5%	-	-	[32]
International delivery costs (per dose)				[32]
ROTARIX	$0.0264	-	-	
ROTAVAC	$0.0249	-	-	
In-country logistics costs, port of entry to central store (per dose)				[32]
ROTARIX	$0.0290	-	-	
ROTAVAC	$0.0173	-	-	
Wastage				MoH
ROTARIX	0.3%	-	-	
ROTAVAC	4.7%	-	-	
Safety boxes	5%	-	-	

### Vaccine efficacy, coverage, and timeliness

Our analysis assumes similar protective efficacy and waning for both vaccines, based on data generated by a recent pooled analysis using all published rotavirus vaccine randomized control trials [23]. We applied pentavalent vaccine coverage rates as reported by the MOH and vaccine coverage timeliness as reported by the latest Palestine MICS [18,24]. Data input values used for vaccine efficacy and coverage are available from Table 1.

### Treatment costs

To estimate treatment costs, we modeled direct medical, direct non-medical, and indirect costs linked to an episode of RVGE. Using data from a study estimating the unit costs of care in public hospitals and primary healthcare centers in Palestine [25], we estimated direct medical costs linked to treating RVGE in inpatient and outpatient settings. To estimate direct medical costs for RVGE care in inpatient settings, we used the unit cost per patient day from this study, which included salaries, operating costs, and overhead in the general surgery ward, the closest fit among available datasets. Based on information received from the MOH, the average length of stay for a child with diarrhea was two days. We further accounted for costs of laboratory tests (CBC, serum electrolytes, and stool analysis) and medicines (two IV Ringer lactate and three packets of oral rehydration solution [ORS]) according to the standard protocol for treating childhood diarrhea in Palestine. Costs for these supplies and drugs were based on unit prices collected from the Palestinian central medical store. We used similar methods to estimate direct medical cost in the outpatient setting, using unit cost per visit in primary health care facilities [25] and accounting for a child receiving three packets of ORS. Based on the standard treatment protocol, we assumed no laboratory tests were done when a child presents with non-severe diarrhea in an outpatient setting.

We also calculated direct non-medical costs based on expert opinion, including expenses for transportation and food for the child and the caregiver. These costs were valued based on local prices and accounted for the setting (inpatient or outpatient) where the child received the care.

We further accounted for the productivity losses of the caregiver, which we valued using the average daily wage in West Bank and Gaza, accounting for unemployment rates and for the distribution of the labor force in each area [26]. We first calculated the daily loss of productivity distinctly for the West Bank and Gaza area. For this we multiplied the average daily wage for employees with formal sector employment by the complement of the unemployment rate (1 minus unemployment rate) as in the equation below.

Rateforemployedpopulation=1−rateforunemployedpopulation

We then calculated an average daily loss of productivity factoring the distribution of the labor population in West Bank and Gaza to estimate the average productivity value per day for Palestine. We assumed two days lost for inpatient care and two hours lost for outpatient care (or 25% of daily productivity value).

Based on standard methods, we allocated direct medical costs to the health system cost while direct non-medical and indirect were allocated to household costs. The sum of health system and household costs is used for the societal perspective. To capture the costs of receiving care in the private sector, we explored a 50% increase of direct medical costs in the sensitivity analysis, based on information shared by MOH staff on the potential magnitude of cost differences between public and private sector. Whereas all children under six years of age in Palestine are covered through governmental insurance and have free access to healthcare in the MOH or facilities run by the United Nations Relief and Works Agency for Palestine refugees (UNRWA), private hospitals also operate, likely capturing a significant share of healthcare utilization.

### Incremental health system costs

We evaluated the incremental financial and economic costs to the health system related to the introduction of the rotavirus vaccine. The data to estimate these incremental costs were obtained through primary and secondary data collection.

#### Incremental supply chain and service delivery costs

To assess supply chain and service delivery costs, primary data collection was carried out in West Bank and Gaza using structured questionnaires in 20 health facilities providing immunization services, 6 health directorates (district offices), and at the central store. We estimated the cost of supply chain and service delivery for all vaccines used in the immunization program and used these data to estimate the incremental economic costs of adding rotavirus vaccine into the immunization program in Palestine. Main cost categories accounted for resources used by the immunization program for cold chain storage (e.g., refrigerators, freezers, cold rooms), transportation of vaccines and immunization supplies, waste disposal, and human resources time for immunization activities.

Financial costs account for actual expenditure on the goods and services purchased. These would include purchases of new cold chain equipment, changes in the vaccine delivery frequency or type of transport used, or new staff hired due to the vaccine introduction. We interviewed MOH collaborators to gather information on whether the introduction of ROTARIX and ROTAVAC vaccines had resulted in any such financial costs.

Economic costs account for the opportunity costs of resources, including existing resources that could have been used for alternative purposes. To estimate cold chain costs, we collected information on the types of cold chain equipment available at each level of the supply chain and combined this with secondary data. The cold chain costs included the annualized capital costs for the existing cold chain and the annual energy costs for operating the cold chain equipment. To estimate the cost per cm^3^ of vaccine storage, total costs for cold chain at each facility were divided by the total vaccine storage capacity of the equipment, adjusted for the utilization rate of the equipment.

To estimate transport costs, we collected information on whether vaccines and immunization supplies were delivered by each facility or collected, the types of vehicles used for the delivery, the expenditures on fuel and maintenance, the number of trips made, and distance traveled. These data were used to estimate transport costs, which include the annualized capital costs for vehicles, and annual expenditures for fuel and maintenance of the vehicles. Except for the five health facilities in Gaza, all other health facilities in the sample had vaccines delivered by the directorate. In order to avoid double-counting of transport costs, we assumed that vaccines are typically delivered by the directorate and so transport costs are only included at the directorate and central or territorial level. We estimated the cost per cm^3^ transported by dividing the total transport costs by the volume of vaccines transported. We adjusted transport costs to account for the fact that vaccines are delivered on the same trip with immunization supplies at the directorate and with other non-immunization program commodities at the central store. Immunization supplies are bulkier, with a typical syringe having a volume of 30 cm^3^ compared to vaccines whose volumes typically range from 1 to 4 cm^3^ per dose in multi-dose vials. We estimated that vaccines would account for about 9% of the volume of commodities transported at the directorate and about 1.4% of the volume transported at the central store.

These estimated cold chain and transport costs per cm^3^ were then multiplied by the volume per dose of ROTARIX (17.12 cm^3^) and ROTAVAC (4.2 cm^3^) to estimate the incremental cold chain and transport cost per dose for each vaccine.

WHO guidelines suggest that an incremental cost assessment should account for 15 minutes per dose of a new vaccine to capture the economic costs of time for vaccine administration and record-keeping [27]. This estimate is consistent with estimates from our analysis. However, we also sought to account for the slight difference in administration time between ROTARIX and ROTAVAC. Based on discussions with some of the nursing staff in West Bank during the field testing of the questionnaires, we understood that ROTARIX is slightly more challenging to administer, due to the larger volume given to the infant (1.5 ml) compared to ROTAVAC (0.5 ml). With ROTARIX, infants tend to regurgitate and there may be need to spend time to make sure the infant gets the full dose; some infants may even need a dose repeated. As a result, we therefore assumed a marginal time differential of 30 seconds for staff time between ROTARIX and ROTAVAC. A larger time differential is possible but the corresponding estimated costs at the health facility would fall within the facility cost uncertainty range examined in the sensitivity analysis.

We estimated the total human resource costs at each level by multiplying the salary for each staff by the percentage of time spent on immunization program-related activities and summing this across the staff at the facility. The information on the time spent on immunization program-related activities was obtained from the costing interviews we conducted, and average salaries by job level/grade were obtained from MOH and UNWRA. We then estimated the cost per minute of staff time at each facility by dividing the total human costs of all staff working in the immunization program at the facility by the estimated total time spent on immunization related activities.

The health workers dispose the vials in the same safety boxes as sharps waste (all medical waste were disposed in safety boxes). To estimate the cost per cm^3^ for waste disposal, we used secondary data provided under the assumption that it costs NIS 5 (~US$1.40) per kilogram to dispose of sharps waste [28]. We estimated the cost of waste disposal assuming 1 liter = 1 kilogram, as per standard metric conversions, and estimated the waste disposal cost per cm^3^ and per dose of each vaccine.

#### Introduction costs

To assess the costs linked to introducing ROTARIX in 2016 and the additional costs linked to switching products in 2018, we conducted key informant interviews within the Department of Public Health within the MOH to identify the activities that occurred with the rotavirus vaccine introduction and identify data sources. Respondents from UNRWA and UNICEF, active partners of the vaccination program, were also interviewed and provided information on the activities conducted and corresponding expenditure data. Introduction costs were mainly collected from invoices and expenditure reports provided by the MOH immunization team and other partners. In line with WHO guidelines for estimating costs of new vaccine introduction [29], we used an incremental costing approach to assess the cost of activities that took place and investments for the addition of the rotavirus vaccine to the routine immunization program. For each activity or investment, we estimated both financial and economic costs. We gathered introduction costs separately for Gaza and the West Bank.

The main cost categories assessed included training of MOH staff and communication materials. While financial costs were retrieved from financial reports and invoices, economic costs were calculated by valuing the staff time spent on training, using information provided by MOH directors on duration of training, number and type of staff trained, and types of training. Introduction costs were collected for the introduction of ROTARIX in 2016 and for the switching from ROTARIX to ROTAVAC in 2018.

In order to combine introduction costs with other recurrent costs (supply chain and service delivery costs), we annualized the figure obtained through secondary data collection. For this process we assumed a 3-year period during which introduction cost benefits last. For annualizing the introduction costs, we performed simple annualization without discounting to annualize the introduction costs for training and communication over a three-year period.

### Vaccine procurement costs

Procurement cost data were collected from the local UNICEF office using reports of each shipment of rotavirus that entered Palestine from late-2015 to mid-2018. For the analysis, we used a different vaccine price for ROTAVAC because of the current uncertainty over the final price that the government will be able to access. In our base-case scenario, we used a price of US$1 per dose of ROTAVAC, the price paid by RVF while the organization was supporting the rotavirus vaccination program [30]. In an alternative price scenario, we used a lower price ($0.85), as offered to Gavi countries [31], as well as a higher price ($1.50) in order to reflect a range of possible prices that the government of Palestine may have to pay. For ROTARIX, we used the price paid at the time the vaccine was used ($4) [32]. In addition to price paid, we also collected information on international handling and transportation, including in-country transportation from port of entry to the central store. We used reported wastage data and a series of additional parameters, including international handling and transportation, to model vaccine and commodities costs as displayed in Table 1.

### Sensitivity analysis

To account for uncertainty in some of the parameters used in the analysis, we ran sensitivity analyses covering a range of more and less favorable scenarios. We looked at a lower and higher rotavirus disease burden, higher and lower vaccine efficacy, higher and lower incremental health system costs, and higher and lower vaccine price for ROTAVAC. Low and high values used in the sensitivity analysis and their reference are available from Table 1.

In addition to sensitivity analysis using different scenarios, we also undertook probabilistic sensitivity analysis on a series of parameters, running the analysis 1,000 times to create a cost-effectiveness acceptability curve for the scenario with ROTAVAC. Parameters included in the probabilistic sensitivity analysis are disease burden, DALY weights, vaccine efficacy, vaccine waning, vaccine price, health system costs per dose, and healthcare costs. Low and high data value for each parameter and distributions used are available in supplementary files.

### Cost-effectiveness threshold

To interpret our results, we used a cost-effectiveness or willingness-to-pay threshold of one times the GDP per capita, $3,095 [33], in line with practice reported by local experts for similar economic evaluations performed by the Palestinian MOH.

## Results

### Treatment costs

We estimated that treating one case of severe RVGE in an inpatient setting has a direct medical cost of $173.85. Direct non-medical costs are $28.04 and indirect costs are $35.59, which add up to a total societal perspective cost of $237.48. Treating RVGE in outpatient setting has a much lower cost, with direct medical costs amounting to $7.63 and a total societal perspective cost of $21.43. S1 Table in supplementary material displays the values resulting from our calculations.

### Incremental health system costs

#### Incremental supply chain and service delivery costs

The introduction of ROTARIX and ROTAVAC vaccines did not result in any new purchase of cold chain equipment at any level of the supply chain. The system also had enough transport capacity to accommodate both vaccine introductions. Because rotavirus vaccines were added into the EPI schedule and the schedule aligns with other vaccines already included in the schedule, no new visits were needed and no new staff were hired. Hence, there were no incremental financial costs for cold chain, transport, or human resources associated with the introduction of either ROTARIX or ROTAVAC. We estimated the incremental economic costs for the supply chain and service delivery to average $2.70 per dose for ROTARIX compared to an average of $2.36 for ROTAVAC (Table 2). The incremental economic costs were larger for ROTARIX than ROTAVAC because of the larger volume per dose. The economic costs per dose were also larger at the lower levels of the supply chain because of the relatively higher fixed costs for cold chain and health worker time spent on service delivery, relative to the volume of vaccines used.

**Table 2:** Incremental economic supply chain costs associated with ROTARIX and ROTAVAC.

	ROTARIX			ROTAVAC—5 dose vials		
Cost category	Average	Min	Max	Average	Min	Max
**Estimated incremental economic costs per dose at the health facility level**						
Cold chain	$0.28	$0.04	$1.41	$0.07	$0.01	$0.34
Waste disposal	$0.02			$0.01		
Human resource	$2.01	$1.53	$2.32	$1.95	$1.47	$2.24
Total	$2.32	$1.59	$3.75	$2.02	$1.49	$2.59
**Estimated incremental economic costs per dose at the directorate level**						
Cold chain	$0.03	$0.0330	$0.05	$0.024	$0.006	$0.091
Transport	$0.022	$0.0138	$0.044	$0.005	$0.003	$0.011
Human resource	$0.30	$0.10	$0.50	$0.30	$0.10	$0.50
Total	$0.35	$0.15	$0.59	$0.33	$0.11	$0.60
**Estimated incremental economic costs per dose at the central store**						
Cold chain	$0.0114	-	-	$0.012	-	-
Transport	$0.003	-	-	$0.003	-	-
Human resource	$0.01	-	-	$0.001	-	-
Total	$0.03	-	-	$0.016	-	-
**Total incremental economic costs per dose costs at all levels of the supply chain**						
Total	$2.695	$1.77	$4.37	$2.362	$1.62	$3.21
**Total incremental economic costs per child**						
Total	$5.39	$3.54	$8.74	$7.09	$4.86	$9.63

#### Introduction costs

Economic costs associated with the introduction of ROTARIX in 2016 amounted to a total of $296,263. This is higher than the total economic cost linked with switching to ROTAVAC in 2018, amounting to $159,524. This is explained by the higher intensity of training activities related to introducing rotavirus vaccine for the first time. Training sessions were longer and targeted a higher number of individuals in 2016. Similarly, fewer communication materials were developed for the switch to ROTAVAC. Because of this cost difference, we elected to apply introduction costs of ROTARIX to both vaccines while doing the cost-effectiveness analysis. This allowed for a fair comparison assuming that if Palestine had introduced ROTAVAC as the first rotavirus vaccine, they would likely face similar introduction costs to what was required for ROTARIX. S2 Table in supplementary materials displays details of the financial and economic introduction costs for both vaccines per area. When adjusted to 2018 US$, annualized and combined as per dose value, the introduction cost per dose was $0.35 for ROTARIX and $0.24 for ROTAVAC.

### Impact and cost-effectiveness

Based on the assumption of similar efficacy for the two rotavirus vaccines, health and economic benefits reported in Table 3 are applicable for both ROTARIX and ROTAVAC. Over ten years, rotavirus vaccination in Palestine has the potential to avert approximately 468,000 non-severe RVGE cases, 101,000 severe RVGE cases, and about 100 deaths. This represents potential savings on treatment costs of approximately $14 million for the health system and approximately $22 million for the society.

**Table 3:** Health and economic outcomes (10 cohorts vaccinated over the period 2016–2025, costs discounted).

**Health outcomes**	
Total non-severe cases <5 yrs averted	468,167
Total severe cases <5 yrs averted	101,112
Total outpatient visits averted	301,148
Total hospitalizations averted	80,889
Total deaths <5 yrs averted	102
DALYs (discounted) averted	3,921
**Health system treatment costs**	
Outpatient visit costs averted	$1,983,909
Hospitalization costs averted	$12,141,801
*Total health system treatment costs averted*	*$14*,*125*,*710*
**Societal treatment costs**	
Outpatient visit costs averted	$5,572,106
Hospitalization costs averted	$16,585,763
*Total societal treatment costs averted*	*$22*,*157*,*869*
**Vaccine program costs**	
With ROTARIX (at $4 per dose)	$19,044,961
With ROTAVAC (at $1 per dose)	$15,510,482
With ROTAVAC (at $0.85 per dose)	$14,826,329
With ROTAVAC (at $1.5 per dose)	$17,790,994
**Cost per DALY averted** (ROTAVAC at $1 per dose)	
ROTARIX compared to no vaccine (health system perspective)	$1,254
ROTARIX compared to no vaccine (societal perspective)	Cost-saving (-$794)
ROTAVAC compared to no vaccine (health system perspective)	$353
ROTAVAC compared to no vaccine (societal perspective)	Cost-saving (-$1,695)
ROTAVAC compared to ROTARIX (health system perspective)	Cost-saving (-$901)
ROTAVAC compared to ROTARIX (societal perspective)	Cost-saving (-$901)

The 10-year vaccination program costs vary depending on which vaccine and price are considered. It ranges from a high of $19 million with ROTARIX at $4 per dose to a low of $14.8 million with ROTAVAC at $0.85 per dose. In our base case scenario with ROTAVAC at $1 per dose, the vaccination program cost is $15.5 million.

As shown in Table 3, the incremental cost-effectiveness ratio from the health system perspective is estimated at $1,254 with ROTARIX and $353 for ROTAVAC compared to no vaccination program. From the societal perspective, both vaccines were cost-saving in our base-case scenario compared to no vaccination program. Assuming a similar health impact of both vaccines and with lower vaccination program costs for ROTAVAC, switching from ROTARIX to ROTAVAC was a cost-saving alternative for Palestine.

### Sensitivity analysis

**Fig 1:**
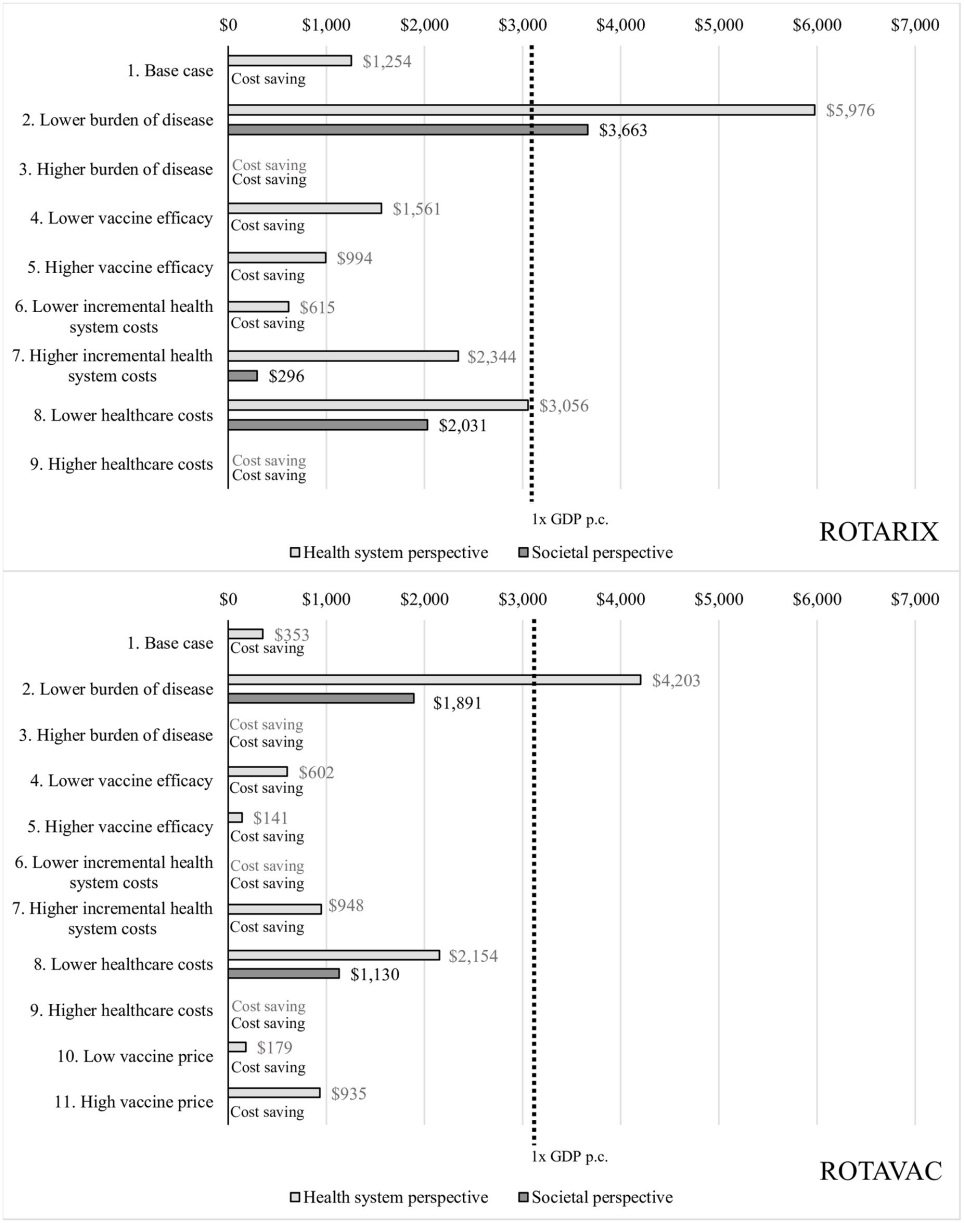
Discounted cost per DALY averted for ROTARIX and ROTAVAC under different scenarios. For each scenario, the first result presented is from the health system perspective and the second result is from the societal perspective.

The different scenarios explored in the sensitivity analyses show that in many of the scenarios, rotavirus vaccination with either vaccine is a cost-saving intervention in Palestine; i.e., rotavirus vaccination is both more effective (averts more DALYs) and costs less than no vaccination. Only when we assume a very low burden of disease was rotavirus vaccine not cost-effective; i.e., where the cost per DALY averted would be above the threshold of one times the GDP per capita. ROTAVAC shows better value for money over ROTARIX in all scenarios analyzed, including when accounting for a higher vaccine price of $1.50 per dose. Disease burden and healthcare costs are the two main drivers of uncertainty. Fig 1 displays the results in terms of discounted cost per DALY averted for all scenarios explored.

**Fig 2:**
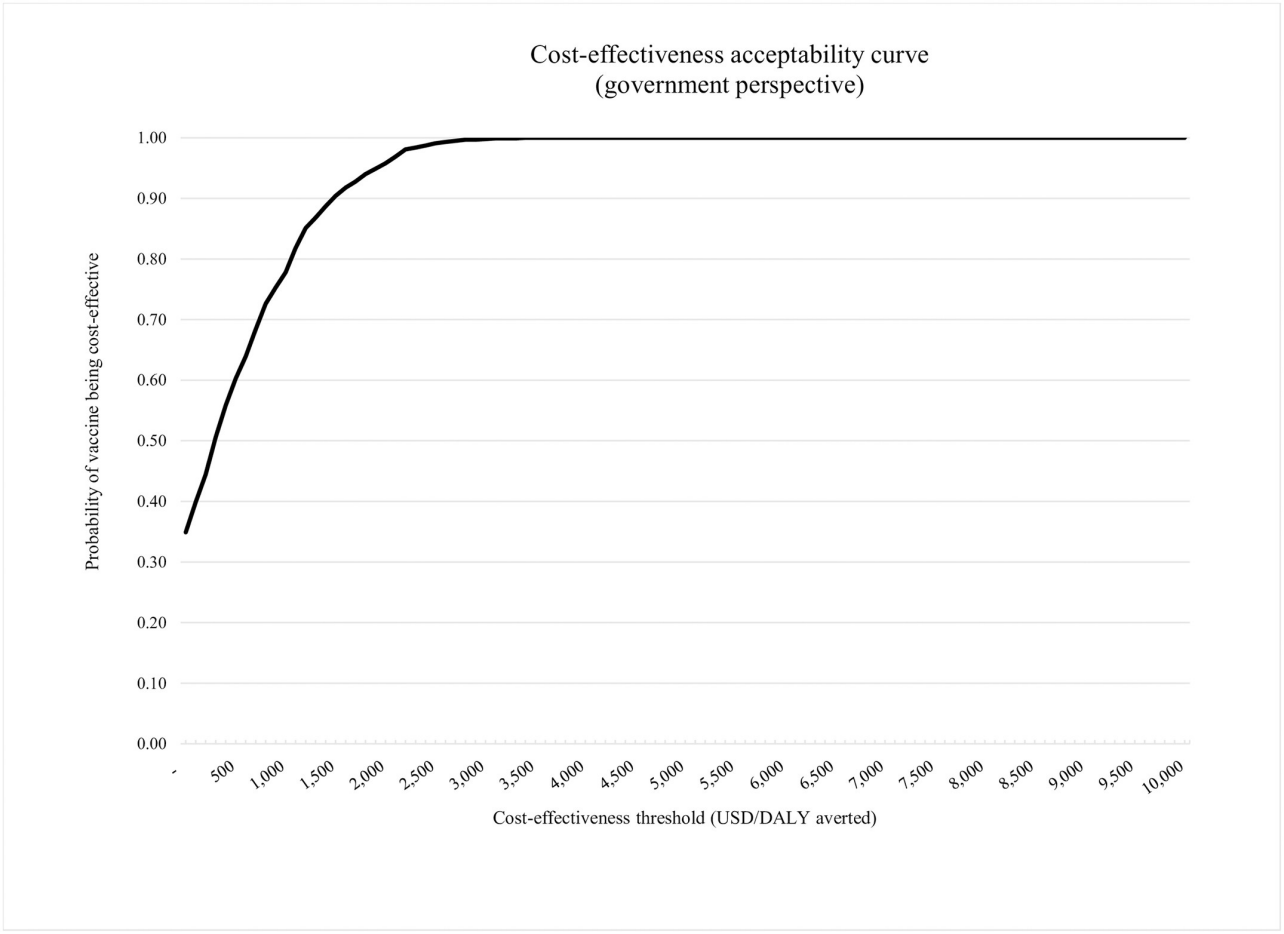
Cost-effectiveness acceptability curve.

From the health system perspective, ROTAVAC has a 90% chance of being cost-effective at a threshold of $1,500 per DALY averted (note that $1,500 is 50% of the GDP per capita). At a threshold of $3,000 per DALY averted (i.e., the threshold used in most of the results reported above), the probability of ROTAVAC to be a cost-effective intervention in Palestine is 100%. The cost-effectiveness acceptability curve displayed in Fig 2 shows the results of the probabilistic sensitivity analysis.

## Discussion

From the health system perspective, rotavirus vaccination with either ROTARIX or ROTAVAC is a cost-effective intervention in Palestine compared to no vaccination, averting a share of the rotavirus disease burden and generating substantial savings on healthcare costs. When accounting for averted healthcare-related costs for households, using either vaccine is a cost-saving intervention, highlighting the economic burden rotavirus represents for households and the health system in Palestine. Other studies conducted in the EMRO region have also found that rotavirus vaccine introduction is cost-effective or cost-saving compared to no vaccination [14,36,37]. When evaluating the switch, ROTAVAC presents an economic advantage over ROTARIX and shifting from ROTARIX to ROTAVAC was a cost-saving option from both the health system and societal perspectives. Savings due to a much lower price per dose for ROTAVAC compared to ROTARIX outweigh any cost disadvantages resulting from having a three-dose rather than two-dose schedule. The price of ROTARIX must fall to approximately $2.75 in Palestine to achieve a cost-effectiveness ratio comparable to ROTAVAC.

Financial costs associated with the introduction of rotavirus vaccination, including costs related to training of health care workers and printing of communication materials, were modest. There were no financial costs incurred for the supply chain and service delivery since the health system had adequate resources to accommodate the rotavirus vaccine introduction and switch. However, when accounting for the opportunity costs of using existing human resources and vaccine storage equipment and transport, economic costs of the rotavirus vaccine introduction and use are much higher. Other studies have estimated incremental costs for rotavirus vaccine introduction of $2.15 in Zambia and $0.54 in Rwanda [34,35]. Differences in estimates may be due to different country contexts, introduction modalities, and methodology used.

While shifting to ROTAVAC, Palestine managed to keep open vial vaccine wastage rates comparatively low (4.7%) for a multi-dose vaccine presentation. This was achieved by increasing session sizes though aggregation. The reported wastage rate in Palestine is much lower than expected for similar multi-dose products, which typically have wastage rates of 25–30% [31,38]. This finding from Palestine shows the feasibility of reaping the benefits attributable to the lower price per dose associated with a multi-dose vial presentation while minimizing the open vial wastage rate.

Palestine was able to smoothly transition from one rotavirus vaccine product to another, even though vaccine price and other programmatic considerations such as the storage conditions, number of doses, and open vial wastage rates of the two vaccines are different. As other countries consider switching between rotavirus vaccine products, the results from Palestine can provide evidence on the cost tradeoffs of these two vaccine presentations and how these tradeoffs impact cost-effectiveness.

This study has several limitations. We were not able to fully leverage local surveillance data to model rotavirus disease burden in the countries. We attempted to collect data for the year before vaccine introduction to inform our estimated rotavirus incidence rates in the absence of vaccination, but found incomplete data sets from the health information system on hospitalized diarrhea cases. The existing data showed that, in 2015, only a share of the hospitals had electronic reports, and paper records from that time were missing. We also experienced difficulties with the lack of standardization in how diarrhea cases were reported. In Gaza, services are provided through the MOH and UNRWA, but until recently, the two providers reported data for different age groups (0 to 3 years vs. 0 to 5 years), not allowing us to generate consistent numbers for children under a single age span. Finally, data on primary healthcare visits for diarrhea were reported for the entire population, not differentiated by specific age groups. We addressed these data limitations by using publicly available global and regional data. To address the uncertainty around these estimates and how well they represent the rotavirus burden in Palestine, we explored fairly large uncertainty ranges in sensitivity analysis.

UNIVAC is a static model which likely underestimates the benefits of vaccination by not accounting for potential herd effects. Others have studied the effect of rotavirus vaccination using dynamic models but findings from different countries tend to show uncertainty around the scale and duration of predicted herd effects [13].

In this analysis, we assumed ROTARIX and ROTAVAC to have similar efficacy, leading to an equivalent health impact. There is currently limited information on ROTAVAC efficacy outside the Indian population, although field effectiveness studies are underway. In India, a randomized, double-blind, placebo-controlled trial showed efficacy levels of 56% in 12-month olds [39].

Of note, a private pediatric hospital in southern Palestine began conducting rotavirus surveillance several years prior to the introduction of rotavirus vaccine and has continued these activities through the transition to ROTAVAC. This monitoring showed a decrease in rotavirus prevalence following introduction of ROTARIX [10]. Our assumption of similar efficacy for the two vaccines may be confirmed in the near future as more recent epidemiological surveillance data become available.

Because of the paucity of data, we do not differentiate vaccine efficacy between severe and non-severe rotavirus disease. Although this may overestimate the impact on non-severe disease, it would remain within the bounds of our uncertainty range and has little influence on results.

To our knowledge, this is the first economic analysis of the use of ROTAVAC outside of India using empirical data. The Palestine experience can be considered by countries in their decision-making processes around rotavirus vaccination, particularly in the context of the availability of other new rotavirus vaccine products. Despite the potential challenges with disease burden or cost data availability for modeling, countries should consider systematically assessing multiple aspects beyond vaccine price when comparing the health and economic value of several products in order to fully account for all characteristics. However, for countries not eligible for Gavi support who may be exploring the financial sustainability of their immunization programs, our analysis suggests that vaccine price is a critical variable and that their choice of vaccine should be re-assessed when new, low-cost products become available.

## Supporting information

S1 Table Treatment costs. (DOCX)

S2 Table Introduction costs with ROTARIX and ROTAVAC. (DOCX)

S3 Table Probabilistic sensitivity analysis assumptions. (DOCX)

S1 Fig Discounted cost per DALY averted for ROTARIX and ROTAVAC under different scenarios (with negative ICER values). (TIF)
