# Supplementary material for: Introduction of rotavirus vaccination in Palestine: An evaluation of the costs, impact, and cost-effectiveness of ROTARIX and ROTAVAC
Source: PLoS One. 2020 Feb 5;15(2):e0228506. doi: 10.1371/journal.pone.0228506 (PMC7001920; doi:10.1371/journal.pone.0228506)
Supplement: S1 Table — (DOCX) [file pone.0228506.s001.docx]

**S1 Table. Treatment costs**

|  | **Health system perspective**  ***Direct medical costs*** | ***Direct non-medical cost*** | ***Indirect cost*** | **Societal perspective**  ***Total*** |
| --- | --- | --- | --- | --- |
| Treatment cost for RVGE inpatient care | $173.85 | $28.04 | $35.59 | $237.48 |
| Treatment cost for RVGE outpatient care | $7.63 | $9.35 | $4.45 | $21.43 |
