# Supplementary material for: Introduction of rotavirus vaccination in Palestine: An evaluation of the costs, impact, and cost-effectiveness of ROTARIX and ROTAVAC
Source: PLoS One. 2020 Feb 5;15(2):e0228506. doi: 10.1371/journal.pone.0228506 (PMC7001920; doi:10.1371/journal.pone.0228506)
Supplement: S2 Table — (DOCX) [file pone.0228506.s002.docx]

**S2 Table. Introduction costs with ROTARIX and ROTAVAC**

| **ROTARIX introduction costs (2016 US$)*** | | | |
| --- | --- | --- | --- |
|  | **West Bank** | **Gaza** | **Total** |
| **Financial costs** | **29,300** | **32,098** | **61,398** |
| Training | 17,650 | 9,861 | 27,511 |
| Communication materials | 11,650 | 22,237 | 33,887 |
| **Economic costs** | **220,098** | **76,165** | **296,263** |
| Training | 208,448 | 53,928 | 262,376 |
| Communication materials | 11,650 | 22,237 | 33,887 |
| **ROTAVAC Switching costs (2018 US$)** | | | |
|  | **West Bank** | **Gaza** | **Total** |
| **Financial costs** | **18,918** | **19,439** | **38,357** |
| Training | 9,710 | 13,556 | 23,266 |
| Communication materials | 9,208 | 5,883 | 15,091 |
| **Economic costs** | **106,765** | **52,759** | **159,524** |
| Training | 97,557 | 46,876 | 144,433 |
| Communication materials | 9,208 | 5,883 | 15,091 |

^*^We elected to apply introduction costs of ROTARIX to both vaccines while doing the cost-effectiveness analysis. This allowed for a fair comparison assuming that if Palestine had to introduce ROTAVAC as the first rotavirus vaccine in the country, they would likely face similar introduction costs to what was required for ROTARIX.
