# Supplementary material for: Introduction of rotavirus vaccination in Palestine: An evaluation of the costs, impact, and cost-effectiveness of ROTARIX and ROTAVAC
Source: PLoS One. 2020 Feb 5;15(2):e0228506. doi: 10.1371/journal.pone.0228506 (PMC7001920; doi:10.1371/journal.pone.0228506)
Supplement: S3 Table — (DOCX) [file pone.0228506.s003.docx]

**S3 Table. Probabilistic Sensitivity Analysis assumptions**

| **Parameter** | **Base case (95% CI)** | **Probability distribution** |
| --- | --- | --- |
| **Disease burden estimates** |  |  |
| RVGE incidence rate <5 years per 100,000 | 10,000 (7,000-14,000)  4,350 (3,259-6,016)  939.5 (444-1,390)  1,421 (555-2,102) | Beta-PERT (mid = regional value accounting for country specific diarrhea treatment seeking behavior, range = reported uncertainty range) |
| Non-severe RVGE visits |  |  |
| Severe RVGE visits |  |  |
| Severe RVGE hospitalizations |  |  |
| Severe RVGE mortality rate <5 years | 2.03 (0.84-4.88) | Beta-PERT (mid = Log transformed mean of 3 sources of country estimates, range = 95% CI) |
|  |  |  |
| **Disability weights for calcualting DALYs** |  |  |
| Percentage of healthy time lost whilst living with the disease |  |  |
| Non Severe RVGE | 18.8% (12.5-26.4) | Beta-PERT (mid = Salomon, GBD 2013, moderate diarhea, range = Salomon, GBD 2013, moderate diarhea, 95% uncetainty interval) |
| Severe RVGE | 24.7% (16.4-34.8) | Beta-PERT (mid = Salomon, GBD 2013, severe diarhea, range = Salomon, GBD 2013, severe diarhea, 95% uncetainty interval) |
|  |  |  |
| **Initial efficacy against RVGE**  (2 weeks after dose administered) | 91.4% (89.8-92.7) | Beta (alpha = 1394·26, beta = 131·74, [A] = 0%, [B] = 100%) |
|  |  |  |
| **Mean duration of vaccine efficacy in months** | 121.9 (81.3-182.4) | Gamma (alpha = 24·01, beta = 5·28) |
|  |  |  |
| **Vaccine price** | $1 ($0.85-$1.5) | Beta-PERT |
|  |  |  |
| **Incremental health system costs per dose** | $2.70 ($1.68-$3.27) | Beta-PERT |
| (Average over 10-year time horizon) |  |  |
|  |  |  |
| **Treatment costs** |  |  |
| Outpatient care health system perspective  Outpatient care visit societal perspective  Inpatient care health system perspective  Inpatient care societal perspective | $7.63 ($3.82-$11.45)  $21.43 ($10.72-$32.15)  $173.85 ($86.93-$260.78)  $237.48 ($118.74-$356.22) | Beta-PERT |
