# Supplementary figures and images for: Introduction of rotavirus vaccination in Palestine: An evaluation of the costs, impact, and cost-effectiveness of ROTARIX and ROTAVAC
Source: PLoS One. 2020 Feb 5;15(2):e0228506. doi: 10.1371/journal.pone.0228506 (PMC7001920; doi:10.1371/journal.pone.0228506)

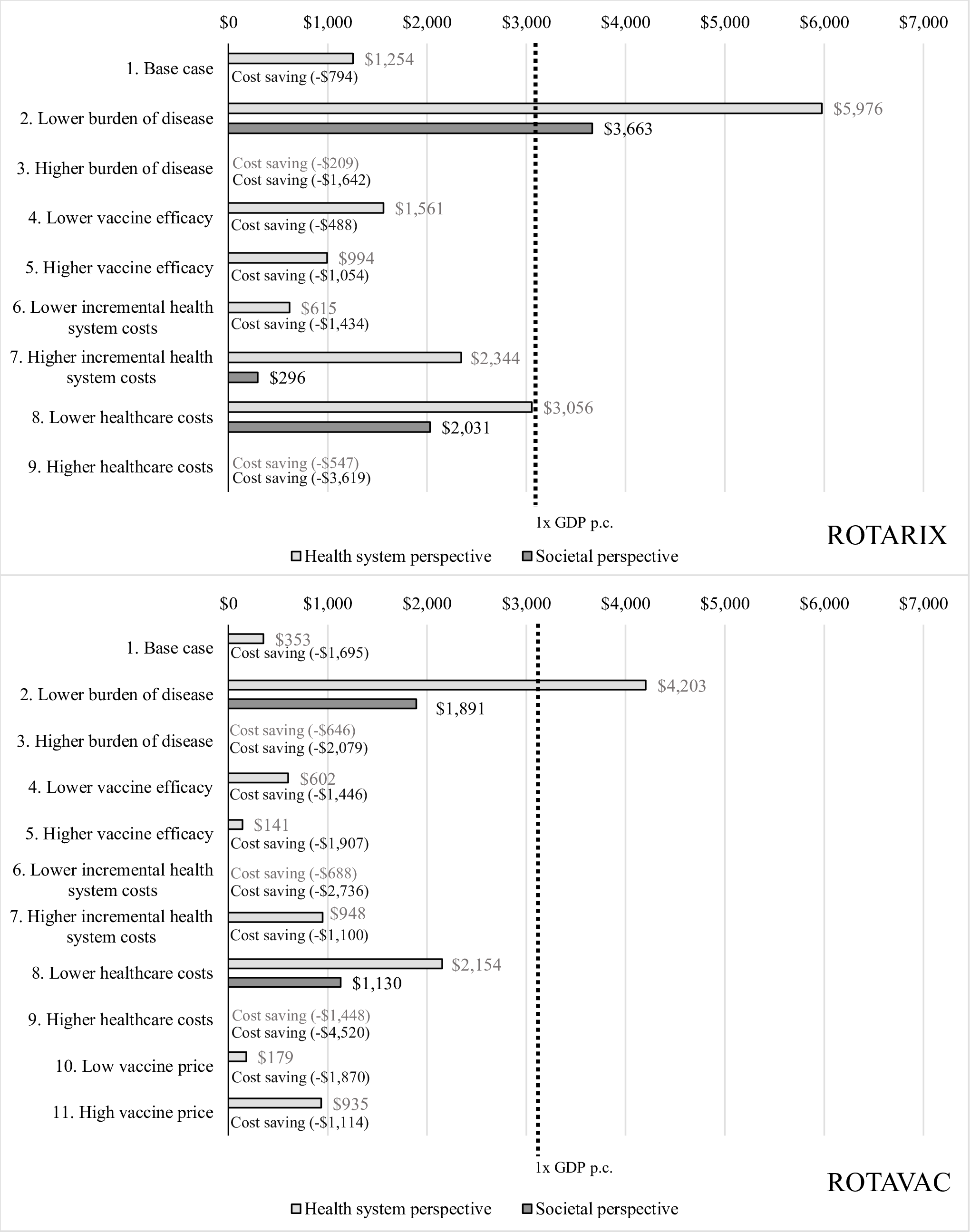

Supplement: S1 Fig — (TIF) [file pone.0228506.s004.tif]
